# Building learning organizational culture during COVID-19 outbreak: a national study

**DOI:** 10.1186/s12913-021-06454-9

**Published:** 2021-05-04

**Authors:** Wadi B. Alonazi

**Affiliations:** Health Administration Department, College of Business Administration, King Saud University, PO Box 71115, 11587 Riyadh, Saudi Arabia

**Keywords:** Learning organization, Culture, Knowledge‐share, COVID-19 outbreak

## Abstract

**Background:**

Hospitals and healthcare institutions should be observant of the ever-changing environment and be adaptive to learning practices. By adopting the steps and other components of organizational learning, healthcare institutions can convert themselves into learning organizations and ultimately strengthen the overall healthcare system of the country. The present study aimed to examine the influence of several organizational learning dimensions on organization culture in healthcare settings during the COVID-19 outbreak.

**Methods:**

During COVID-19 crisis in 2020, an online cross-sectional study was performed. Data were collected via official emails sent to 1500 healthcare professionals working in front line at four sets of hospitals in Saudi Arabia. Basic descriptive analysis was constructed to identify the variation between the four healthcare organizations. A multiple regression was employed to explore how hospitals can adopt learning process during pandemics, incorporating several Dimensions of Learning Organizations Questionnaire (DLOQ) developed by Marsick and Watkins (2003) and Leufvén and others (2015).

**Results:**

Organizational learning including system connections (M = 3.745), embedded systems (M = 3.732), and team work and collaborations (M = 3.724) tended to have major significant relationships with building effective learning organization culture. Staff empowerment, dialogues and inquiry, internal learning culture, and continuous learning had the lowest effect on building health organization culture (M = 3.680, M = 3.3.679, M = 3.673, M = 3.663, respectively). A multiple linear regression was run to predict learning organization based on the several variables. These variables statistically significantly predicted learning organization, *F* (6, 1124) = 168.730, *p* < .0005, *R*^2^ = 0.471, (*p* < .05).

**Discussion:**

The findings concluded that although intrinsic factors like staff empowerment, dialogues and inquiry, and internal learning culture, revealed central roles, still the most crucial factors toward the development of learning organization culture were extrinsic ones including connections, embed system and collaborations.

**Conclusions:**

Until knowledge-sharing is embedded in health organizational systems; organizations may not maintain a high level of learning during crisis.

## Background

Having a primary role in restoring and maintaining wellbeing, healthcare systems largely depend on organizations, people, and their competencies [1]. Particularly, performance of healthcare systems varies based on the structure, process and expected outcomes [2]. On a micro level, healthcare organizations such as hospitals and medical institutions remain at the nucleus of the healthcare system regardless of the type of funding and structure and the provided services to the general population [1]. The key indicator of hospital performance is the safe and consistent services provided to patients by medical and non-medical staff [3]. Therefore, coordination and communication within and between the teams is a must to develop cohesive functioning essential to provide high quality of medical care [4]. Healthcare organizations are always bound to modify their functioning due to dramatic changes in prevalence of diseases and pandemics without compromising the quality of services [5]. Acquiring advanced technologies and creation and utilization of contemporary knowledge can help healthcare organizations to perform well.

Learning organization is considered as a cumulative phenomenon facilitating personal and professional growth of individuals and teams. It also develops collective learning in an organization that leads to enhance individual as well as organizational performance [6]. Indeed, learning organization improves the efficiency and effectiveness of an organization through shared knowledge [7]. An organization that practices continuous learning of employees, bound to transform itself where employees continuously create, acquire, and share knowledge is called learning organization [8]. Learning organization and organizational learning has been used interchangeably in previous literature. However, there is a thin line which differentiates the two. According to Preskill and Torres learning organization focuses on characteristics, principles, and systems whereas organizational learning emphasizes on the process of learning [9]. However, in each level, individuals are the main agents that involve in learning organization or organizational learning and bring substantial changes. Ultimately, organizational learning is a process through which an organization develops new knowledge and understands from routine experiences of the employees. Organizational learning has the potential to change the behavior of employees and improve the organizational capability on policy and practical levels [10].

Learning organization is the first step towards obtaining dynamic knowledge that brings change among employees, whereas in the context of a learning organization, knowledge is acquired and shared among employees via a system that develops capacity to improve performance [10]. Generally, learning organization is influenced by contextual factors such as culture. An organization that regards learning as absolutely critical for its business success is considered well-equipped with knowledge culture [11]. Organizational culture is the characteristic of an organization which manifests the sharing of common values and beliefs among its employees [12]. Thus, learning organization culture develops skills within an organization to create, acquire, and transfer knowledge and enhance positive behavior to follow new medical practice or guidelines [13].

As changing environment forces every healthcare organization to enhance quality and safety, practice of learning organization can improve the knowledge and skills of medical staff and guide them to find better ways to work effectively [14]. Collective learning among small groups or teams could lead to standard performance of healthcare organization through shared knowledge and better understanding among teams [15]. In the context of healthcare services, members of the teams may convert their knowledge in actions and later evaluate actions on evidence based practice associated with contemporary guidelines [7]. Reay and others argued that in healthcare services, physicians and managers first choose the correct knowledge from existing ones and adapt the knowledge to solve problems and find solutions at hand. This process can help in managing conflicts between management and medical professionals. The process of learning in healthcare is time consuming; yet it provides the precise way to cope with medical crises [16].

### Components of Health Learning Organization culture

The early studies on learning organization focused on five factor model developed by Senge including systems thinking, personal mastery, mental models, building a shared vision, and team learning [17]. Gomes and Wojahn conceptualized learning organization on the basis of four components as experimentation, interaction, risk, and dialogue, but concluded that learning organizations had the capability to improve innovation performance [18]. Additionally, Halim and others included three components of learning organization as information acquisition, information interpretation, and behavioral and cognitive and initiated an imperative role of these factors in innovation culture and performance [19].

In the context of healthcare organization, Leuven and colleagues developed seven dimensions that measure organizational learning in low resources healthcare settings [4]. These seven dimensions are continuous learning, dialogue and inquiry, team learning and collaboration, embedded systems, empowerment, systems connections, and strategic leadership. Previous studies on organizational learning in healthcare settings included all top, middle, and lower level employees as part of the study [4, 20, 21]. However, a little is focused on the role of healthcare professionals during crisis to create an environment of organizational learning. During crisis, knowledge management is complex due to the fact that various networks apply different strategies like centralization and other organizational structure like independency [22]. Therefore, the study excluded the dimension of strategic learning in conceptualization of strategic learning mainly focus on support of leadership on learning and leadership models.

### Promoting organizational learning during crisis

Creating, retaining, and transferring useful knowledge are key elements when health institutions incorporate a new model for corporate learning and development [23]. During crisis, medical institutions take a few effective staff in formal committee to accelerate the process ahead of other activity, projecting the less risky roads. Building health protocols, ensuring its effectiveness, as well as revealing the practice among healthcare professionals was a major goal to deal with COVID-19. Again, what promotes organizational learning is the nature of the emergency, which is in this case a pandemic issue [24].

**Fig. 1:**
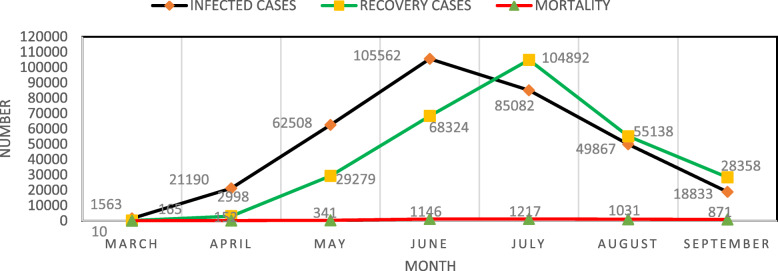
Infected, recovery, and mortality cases during the study period in Saudi Arabia

Though the Saudi government issued instructions to lockdowns, self-isolation, and social distancing, such instructions were initiated based on international learning system, mostly from the World Health Organization (WHO), as well we as the internal experience [25]. However, MOH has taken effective steps in almost each hospital to prevent the spread of the viruses. The MOH initiated on online medical consultations and many activities to learn from this anise. Figure 1 shows the number of new, infected, and death cases in Saudi Arabia during the study period.

### Saudi healthcare system

In Saudi Arabia, the healthcare system is at cross roads where the old publicly funded system, regulated mainly by the government, is now under transition period to be privatized with less power of the government [5]. Almost two-thirds of health provisions are funded by the government, under the regulation of the Ministry of Health. Semi-government agencies like Ministry of Defense, Ministry of Guard and Ministry of Interior contribute also in funding their health settings [26]. Despite such services by the government, private sector and university hospitals contribute slightly in operating the Saudi healthcare system. Again, social reform is an integral part to effective health improvement [27]. As the government is under heavy transition periods by the new health leadership, it is expected the funding portion is flipped within decades [5, 28]. As a result of each provision having different entities and priorities, a chasm now may exist between knowledge and practice within each entity [29].

Having several healthcare providers, the learning organization culture of each entity across the country has not been well-studied [30]. However, learning organization is necessary to implement the strategies that could benefit the organization. In addition, working productivity is also dependent on improved working efficiency and environment that are by products of organization learning [31]. Understanding learning organization culture in such contexts would enhance the resilience of the Saudi system and may enable it to better absorb the adverse effects of the economic and political shocks, especially under such transition periods.

## Methods

This is a cross-sectional study where the overriding objective was to explore some domains associated with building effective organization learning culture during crisis.

### Tool

Consisting of 21 items, the abbreviated form of Dimensions of Learning Organizations Questionnaire (DLOQ) developed by Marsick and Watkins [32] and Leuve was utilized in this study. The purpose of using this tool was simply because it possessed construct validity and sound reliability. Three adequate measurement items (individual, group, and organization) for each dimension included in this study.

### Data Collection

During March to September 2020, data were collected from four major healthcare providers including Ministry of Health (MOH), Teaching University Hospital (TUH), Semi Government (SGH), and Private Hospitals (PH). During partial lockdown, an on line survey was sent to some corporate communication departments to liaise the survey, after obtaining the IRB. The unit of analysis was the first line of healthcare workers who directly deal with COVID-19 cases. The judgmental sampling method was used to identify the eligible respondents and data were collected through electronic mails.

### Procedure and sampling

This study used G*power software to calculate the minimum sample size as recommended by Hair and others for PLS-SEM analysis and found minimum sample of 146 was adequate as maximum six predictors pointing at one endogenous variable [33]. Medium effect size and 0.95 power of the model were set for calculation. The sample size of 1131 of the study satisfied the condition of minimum sample size.

### Measurement

All items of the constructs were adapted from the existing literature and were slightly modified to fulfill the objective of the study [4, 32]. All the items were measured on a five-point Likert scale that ranged from (1) strongly disagree to (5) strongly agree.

## Results

Out of 1500 assigned emails, only 1131 responses returned as they were used for final data analysis. In regard to learning environment, government, and semi-government healthcare settings tend to provide better learning environment than private hospitals. As, from the total respondents of government, 95.6 %; semi-government, 96.1 %; and university hospitals, 94.3 % responded that the hospital in which they work is a real learning organization. However, in case of private hospitals 88.9 % of the total respondents confirmed that the hospital is a learning organization as shown in Table 1.

**Table 1:** Responses about the possibility of having organization as a place of learning

Is this hospital considered a learning organization?		Hospital Type^a^									
		MOH		TUH		SGH		PH		Overall	
		*n*=274	%	*n*=244	%	*n*=333	%	*n*=280	%	*n*=1131	%
**Male**	Yes	131	48	121	50	174	52	141	50	567	50.1
	No	7	2	7	3	8	2	16	6	38	3.4
**Female**	Yes	131	48	109	45	146	44	108	39	494	43.7
	No	5	2	7	3	5	2	15	5	32	2.8

^a^*MOH* Ministry of Health, *TUH* Teaching University Hospital, *SGH* Semi-governmental Hospital, *PH* Private Hospital

The representation of the respondents was almost equally distributed from all four categories regarding their work nature in the hospitals i.e., government hospitals (24.2 %), university hospitals (21.6 %), semi-government hospitals (29.4 %), and private hospitals (24.8), as shown in Table 2.

**Table 2:** The demographic characteristics of the participants and their career background

The nature of the respondents’ work		Hospital Type^a^								
		MOH			TUH		SGH		PH	
		*n*=274		%	*n*=244	%	*n*=333	%	*n*=280	%
Yes	Clinical	M	49	18	77	32	62	19	62	22
		F	42	15	74	30	42	13	49	18
	Administrative	M	62	23	33	14	94	28	71	25
		F	68	25	27	11	74	22	50	18
	Both	M	20	7.3	11	4.5	18	5.4	8	2.9
		F	21	7.7	8	3.3	30	9	9	3.2
No	Clinical	M	2	0.7	5	2	2	0.6	9	3.2
		F	0	0	6	2.5	1	0.3	8	2.9
	Administrative	M	5	1.8	2	0.8	4	1.2	4	1.4
		F	4	1.5	1	0.4	3	0.9	5	1.8
	Both	M	0	0	0	0	2	0.6	3	1.1
		F	1	0.4	0	0	1	0.3	2	0.7

^a^*MOH* Ministry of Health, *TUH* Teaching University Hospital, *SGH* Semi-governmental Hospital, *PH* Private Hospital, *Y* Yes, *N* No, *M* Male, *F* Female

As shown in Table 3, teaching university hospital (TUH) indicated the highest level in building learning organization among the rest, with the highest level in effective system connection and the lowest in continuous learning process. The lowest level in building learning organization was the private Health Hospitals (PH), in the highest score of system connections and the lowest in building internal continues learning and equally building the culture of learning (M = 3.13, and M = 2.95, respectively).

**Table 3:** Results of descriptive analysis including the mean of each setting

Latent Construct/Item	MOH (*n* = 275)	TUH (*n* = 244)	SGH (*n* = 333)	PH (*n* = 280)
Continuous Learning (CL)	3.12	4.11	4.37	2.95
Dialogue and Inquiry (DI)	3.00	4.37	4.27	3.03
Team Learning and Collaboration (TC)	3.16	4.35	4.32	3.02
Embedded Systems (ES)	3.27	4.38	4.25	3.00
Empowerment (EM)	3.01	4.35	4.27	3.05
Systems Connections (SC)	3.15	4.42	4.26	3.13
Learning Organization Culture (SL)	3.07	4.38	4.26	2.95
Over all	3.11	4.34	4.29	3.02

In Table 4, a correlation matrix was constructed to measure the strength between the intrinsic and extrinsic variables when building effective learning organization during crisis.

**Table 4:** Correlation matrix to measure the strength between the intrinsic and extrinsic variables

Domain/ Statistical Value		SL	CL	DI	TC	ES	EM	SC
SL	P	1						
	Sig.							
CL	P	0.507^a^	1					
	Sig.	0.000						
DI	P	0.523^a^	0.547^a^	1				
	Sig.	0.000	0.000					
TC	P	0.535^a^	0.483^a^	0.580^a^	1			
	Sig.	0.000	0.000	0.000				
ES	P	0.539^a^	0.472^a^	0.523^a^	0.592^a^	1		
	Sig.	0.000	0.000	0.000	0.000			
EM	P	0.540^a^	0.486^a^	0.548^a^	0.587^a^	0.526^a^	1	
	Sig.	0.000	0.000	0.000	0.000	0.000		
SC	P	0.572^a^	0.487^a^	0.553^a^	0.565^a^	0.505^a^	0.584^a^	1
	Sig.	0.000	0.000	0.000	0.000	0.000	0.000	

^a^ Correlation is significant at the 0.01 level (2-tailed)

A multiple linear regression (MLR) was constructed to model the linear relationship between the explanatory variables and building effective organizational culture in the assigned settings. As shown in Table 5, a significant regression equation was found (*F* (6, 1124) = 168.730, *p* < .0005, *R*^2^ = 0.471).

**Table 5:** Summary of MLR analysis for variables predicting building effective leaning organizational culture

Model	Unstandardized Coefficients		Standardized Coefficients	t	Sig.
	B	Std. Error	Beta		
Constant	0.340	0.107		3.179	0.002
CL	0.152	0.028	0.151	5.459	0.000
DI	0.096	0.031	0.094	3.117	0.002
TC	0.100	0.032	0.097	3.099	0.002
ES	0.180	0.029	0.177	6.119	0.000
EM	0.136	0.031	0.134	4.427	0.000
SC	0.235	0.031	0.224	7.545	0.000

We can estimate the model to build effective learning organization during COVID-19 crisis, knowing the values for the six variables, by using the regression model:

SL = 0.340 + 0.152 (CL) + 0.096 (DI) + 0.100 (TC) + 0.180 (ES) + 0.136 (EM) + 0.235 (SC).

All six variables are significant predictors of learning organization. *P*-values < 0.05 and even 0.01 for all the variables. The result explained that the internal system and connections were superior, compared to other methods. As shown, the *R*^2^ value for learning organization culture of 0.475 suggests that 47.5 % of the variance of learning organization culture can be explained by six independent variables. The structural model represents the assumed relationship between some variables [33]. The results revealed that continuous learning, dialogues and inquiry, team learning and collaboration, embedded systems, empowerment, and systems connections all have significant positive relationship with learning organization culture.

## Discussion

The aim of the study was to examine the factors influencing building effective learning organization culture in various healthcare. Having theoretical and practical implications, this study bridges the gap in literature on learning organization culture in healthcare settings. Besides, study findings guide top management of the hospitals and policy makers to develop policies and guidelines based on organization learning that create cohesive work environment among various departments of hospitals to provide quality services to patients. Becoming a learning organization is complex and provider-based specific. Unlike process and outcomes indicators, the structural indicators have influence on formulating effective health learning organization culture [34].

First, structural indicators including system connections, embedded system and team work were reported to have a major influence on learning organization. Indeed, the structural components of the health organization have the strongest relationship in formulating health learning organization culture. Systems connections explain that an organization must observe a problem from different aspects and encourage employees to engage across the organization and with outside environment to bring solutions [17]. Unlike European health settings, hospitals in Asian countries, generally, lack practice of engaging the general population to develop new ideas and learning [35, 36].

Collaboration with community welfare organizations, local health agencies, and health consultants can bring advanced knowledge to hospitals and guide in implementing strategies to overcome challenges faced by the hospitals. Team learning is a situation where individuals think together, share experiences, knowledge and skills to do the things in better way [17]. Hospital management and policy makers could promote a culture of engagement of employees within an extrinsic-intrinsic environment and share new knowledge across the organization for continuous learning. Acquired knowledge incorporated from outsiders but embedded in the working systems, practices, and structures can be used and shared to improve performance [32]. In healthcare settings, the learning acquired from the new knowledge should be deep rooted and become part of daily operations of the hospitals.

Empowerment, dialogue and inquiry, internal and continues learning philosophy were also positively related to learning organization culture. Empowerment is a process in which employees of every level take part in collective decision making and accountability. This practice develops motivation among employees to acquire new knowledge so that they can take better decisions [32]. However, in healthcare settings empowering every employee to a level where s/he becomes a part of collective decision making is a huge challenge. Healthcare systems all across the world are predominantly governed by bureaucracy and hierarchical structure with set rules and operating procedures and left no room for many employees to be a part of decision making [37, 38]. Employees, both medical and non-medical, remain excluded in decision-making but bound to implement the instruction and guidelines with high perfection. Policy makers and top administration must ensure the inclusion of hospital employees of every level in collective decision making so that they can also become part of learning and contribute to growth of healthcare system. Dialogues and inquiry are the reasoning skills to express views and the capacity to listen and inquire about the views of others [32]. Logical reasoning and dialogues open space for critical thinking and bring logical and appropriate solutions to different situations.

Continuous learning was also found strongly related to learning organization culture. In healthcare services, skills and knowledge can easily be outdated due to evolvement of technology and procedures. Therefore, medical and non-medical staffs must engage in continuous learning for their self-satisfaction and overall quality of healthcare services. However, researchers argued that continuous learning at individual level is important but not sufficient to improve performance unless and until not embedded in systems [32]. Hospital management may ensure that learning is not limited to individuals only and installed as part of systems so that teams and individuals can use it uninterruptedly.

Internal learning indicators were among the least important aspects to develop culture of learning in hospitals and healthcare institutions. However, in most of the Asian work settings expressing views openly and freely is unwelcoming among colleagues and superiors. This is again mainly due to bureaucracy and hierarchical nature of functioning in healthcare systems [22]. Giving voice to every individual to express their reasoning and logical thinking in healthcare institutions will open space to bring in new ideas and contribute to the culture of learning. Providing satisfactory services to patients requires team work and involves teams and individuals of different expertise. Patient visits to hospitals usually come in contact with employees work in different medical and non-medical departments. Employees that come in contact with patients should receive new knowledge and instill it into the working systems. However, many developing healthcare systems face the problem of communication and coordination gap between different departments of the hospital [4, 39]. A culture should be developed within the hospital where systems ingrained with new knowledge are well connected so that learning can be shared across the organization to enhance service quality.

Contextually, caution should be given to generalize the study findings in healthcare systems of other developing countries due to situational and cultural differences. Furthermore, the use of non-probability sampling to collect the data due to the unavailability of sampling frame is another limitation of the study [5]. Future studies should utilize similar variables and test their relationships in different healthcare settings and compare the findings of this study and observe the effect of situational factors and culture on learning organizational culture.

The findings of this current study and the existing body of evidence have approved that there are some associations between effective learning organization and certain extrinsic organizational practices. Indeed, embedded system like linkage of medical episodes, in medical and non-medical fields, have shown a positive impact among the sample in increasing knowledge-environment. Addition, building strong reliable health information system, especially for decision making throughout the hospital would increase the chances of knowledge transfer among health practitioner. The absence of building health learning organization may promote ineffective performance within a holistic healthcare system.

## Conclusions

Learning culture can become a guiding tool for organizations to improve skills and knowledge of individuals and teams and develop a culture to work together and deliver quality services. Policy makers and the top leadership should work towards creating a sense of shared purpose among medical and non-medical staff at middle and lower level management. Well-connected systems embedded with learning culture will help to build effective relationships, coordinated actions and the reflections that strengthen the desirable practices in healthcare while correcting structures, procedures, and assumptions. In simple, leaderships of healthcare organizations can create a continuous learning environment.

This study demonstrated the usefulness of implementation of organizational learning practices based on several dimensions that can lead to transfer hospitals and healthcare institutions into learning organization during crisis.

## Data Availability

All data are available based on academic purposes through the corresponding author (waalonazi@ksu.edu.sa).

## Abbreviations

SA: Saudi Arabia
MOH: Ministry of Health
TUH: Teaching University Hospital
SGH: Semi-governmental Hospital
PH: Private Hospital
CL: Continuous Learning
DI: Dialogue and Inquiry
TC: Team Learning and Collaboration
ES: Embedded Systems
EM: Empowerment
SE: Systems Connections
SL: Learning Organization Culture
MLR: Multiple Leaner Regression
